# Procalcitonin Clearance for Early Prediction of Survival in Critically Ill Patients with Severe Sepsis

**DOI:** 10.1155/2014/819034

**Published:** 2014-02-24

**Authors:** Mohd Basri Mat Nor, Azrina Md Ralib

**Affiliations:** Department of Anaesthesiology and Intensive Care, Kulliyyah of Medicine, International Islamic University Malaysia, Jalan Hospital Campus, 25000 Kuantan, Pahang, Malaysia

## Abstract

*Introduction*. Serum procalcitonin (PCT) diagnosed sepsis in critically ill patients; however, its prediction for survival is not well established. We evaluated the prognostic value of dynamic changes of PCT in sepsis patients. *Methods*. A prospective observational study was conducted in adult ICU. Patients with systemic inflammatory response syndrome (SIRS) were recruited. Daily PCT were measured for 3 days. 48 h PCT clearance (PCTc-48) was defined as percentage of baseline PCT minus 48 h PCT over baseline PCT. *Results*. 95 SIRS patients were enrolled (67 sepsis and 28 noninfectious SIRS). 40% patients in the sepsis group died in hospital. Day 1-PCT was associated with diagnosis of sepsis (AUC 0.65 (95% CI, 0.55 to 0.76)) but was not predictive of mortality. In sepsis patients, PCTc-48 was associated with prediction of survival (AUC 0.69 (95% CI, 0.53 to 0.84)). Patients with PCTc-48 > 30% were independently associated with survival (HR 2.90 (95% CI 1.22 to 6.90)). *Conclusions*. PCTc-48 is associated with prediction of survival in critically ill patients with sepsis. This could assist clinicians in risk stratification; however, the small sample size, and a single-centre study, may limit the generalisability of the finding. This would benefit from replication in future multicentre study.

## 1. Introduction

Serum procalcitonin (PCT) has been studied previously as a promising diagnostic biomarker of sepsis. It has been shown to potentially help in the diagnosis of severe bacterial infections, evaluation of sepsis severity, assessment of the appropriateness of antibiotics or source control strategy, and tailoring of antibiotic therapy [1, 2]. Recent review articles concluded that PCT guided algorithm reduced duration of antibiotic therapy in septic critically ill patients without compromising clinical outcomes [3–5]. The degree of induction of PCT is associated with the severity of systemic infection in sepsis and in the presence of organ dysfunction (severe sepsis and septic shock) [6–10]. Thus PCT may also have some prognostic value. However, its predictive value for survival is not well established. The initial absolute value of PCT concentration in sepsis is not always associated with patients' prognosis. Most studies have shown that it is not possible to predict the prognosis based on high levels of PCT [11–14].

Intensive care unit (ICU) mortality has been shown to be associated with sustained high PCT levels, suggesting that dynamic change of PCT may be a more valuable biomarker [15–17]. Dynamic approach of assessing the biomarkers may give more information on the survival of the patients. A concept of procalcitonin clearance (PCTc) has been introduced in a pilot study as a tool for monitoring the evolution of PCT levels during severe sepsis [18, 19]. PCT clearance measures the relative changes in PCT to the baseline PCT and is postulated to be a better predictor of outcome.

We aimed to prospectively evaluate the diagnostic performance of PCT for sepsis and its predictive performance for mortality. Additionally, we aimed to assess the predictive utility of dynamic changes of PCT and PCT clearance within the first 3 ICU days in a subcohort of patients with severe sepsis and septic shock. In this study, we hypothesise that PCT measured on ICU admission is associated with sepsis, and its changes over 48 hours is associated with mortality.

## 2. Methods

This was a single centre, prospective observational study in the 12-bed mixed medical and surgical ICU of Hospital Tengku Ampuan Afzan, Kuantan, Malaysia. The study was approved by the local ethics committee and registered under the Malaysian National Medical Research Register (NMR-11-1102-9248, https://www.nmrr.gov.my). Systemic inflammatory response syndrome (SIRS), sepsis, severe sepsis, and septic shock were defined according to American College of Chest Physician/Society of Critical Care Medicine (ACCP/SCCM) [20, 21]. Adult patients of more than 18 years old, who fulfilled SIRS definition, were recruited into the study after consent was obtained. Patients who received any antimicrobial treatment for more than 24 hours before the first PCT analysis taken were excluded. A sample size of 98 patients was needed to achieve the relevant anticipated AUC, that is, 0.7, at a significance level of 5%, power of study of 80%, precision of 10% on either side, and 20% dropout rate. This is based on previous study by Castelli et al. [6] who showed that the ratio of prevalence of sepsis to noninfectious SIRS, *k,* is 0.21 (15/71). This was calculated by using formula from Kumar and Indrayaran [22]. The clinicians were blinded to the PCT levels when caring for patients; blood samples were analysed at the end of the study period.

The intensive care specialist completed a questionnaire for each patient on day 1 and day 3 to define clinical suspicion of sepsis. Patients were grouped into sepsis if there was clinical suspicion of infection with or without positive culture; otherwise, they were grouped into noninfectious SIRS. Severe sepsis was referred to sepsis induced hypoperfusion or organ dysfunction. Organ dysfunction was based on the presence of at least one organ failure as defined by the SOFA score. Septic shock as sepsis induced hypotension persisting despite adequate fluid resuscitation. Sepsis induced hypotension was defined as a systolic blood pressure (SBP) < 90 mmHg or mean arterial pressure (MAP) < 60 mmHg. All septic patients received empirical and culture-guided antimicrobial treatment according to local guidelines. They were treated based on recommendations from the current Surviving Sepsis Campaign, modified to meet the most updated evidence from the literature [23].

Daily serum concentrations of PCT were measured during the first 3 days. The first blood samples were drawn within 24 hours of admission or not later than 24 hours after the first dose of antimicrobial agents administered (baseline PCT). Then their levels were repeated after 24 and 48 hours later. The samples were centrifuged and stored at −80°C for later analysis. Procalcitonin was measured by BRAHMS Kryptor compact assay (Henningsdorf, Germany), the most sensitive and highly precise plasma PCT measurement using time-resolved amplified cryptate emission (TRACE) technology assay with the quantitative result in 19 minutes. It is an ultrasensitive PCT measurement with analytical sensitivity of 0.02 ng/mL and improved functional assay sensitivity of 0.06 ng/mL.

Procalcitonin kinetics are expressed as Delta PCT (ΔPCT) concentrations which is the difference between the subsequent and baseline measurement. Delta 24 and 48 hours PCT (ΔPCT-24 and ΔPCT-48) are calculated as Day 1-PCT concentrations minus PCT measured in the following 24 hours (Day 2-PCT) and 48 hours (Day 3-PCT), respectively. The delta values are negative with increasing concentrations and positive with decreasing concentrations. Of interest is how much does the PCT concentration changes in relation to the baseline PCT. To investigate this, PCT clearance at 24 and 48 h (PCTc-24 and PCTc-48) is calculated as percentage of ΔPCT-24 and ΔPCT-48 over Day 1-PCT. The patients were also grouped according to the level of change of PCTc: patients with PCTc > 30% (Group 1) and patients with PCTc < 30% (Group 2). This level of clearance was chosen following the previous paper by Guan et al. and Claeys et al. which defined significant change as 30% increase or decrease in the initial PCT measurement [24, 25]. Baseline Simplified Acute Physiology II Score (SAPS II), baseline Sequential Organ Failure Assessment (SOFA) score, primary source of infection, culture results, ICU, and hospital mortalities were recorded. Clinical parameters such as body temperature, heart rate, white cell count (WCC), and clinical signs of infection were recorded on admission and daily for 3 days.

### 2.1. Statistical Analysis

Statistical analysis was performed using PASW version 18.0 (IBM, Somers, NY, USA) licensed to the IIUM. Results are presented as mean ± SD for normally distributed variables or median (interquartile range) for nonnormally distributed variables. Comparison of variables between the two groups was analyzed using the independent *t-*test for normally distributed variables or the Mann-Whitney test for nonnormally distributed variables. Categorical variables were compared with Chi-Square test. The diagnostic and predictive performance of PCT, ΔPCT, and PCT clearance were assessed by the area under the curve of receiver operating characteristic curve (AUC) of the sensitivity over 1-specificity. Survival analysis was analysed using Kaplan-Meier and Cox regression analysis. The hazard ratios were adjusted to the SAPS II and SOFA scores. These two variables showed a univariate association with mortality with *P* of less than 0.05. AUC and HR are presented with 95% confidence interval.

## 3. Results

A total of 95 patients were included in the study between July 2011 and June 2012. Of this, sixty-seven patients (71%) had sepsis. All patients with sepsis had at least one organ failure and hence were classified as having severe sepsis. Of these, 29 (43.3%) had septic shock as defined as those with cardiovascular SOFA score of 3 or 4.  Table 1 compares the baseline demographic, and clinical characteristics between patients. Patients with sepsis were sicker, with higher SAPS II and SOFA scores than those with noninfectious SIRS.

### 3.1. Diagnostic and Predictive Performance of PCT

**Figure 1:**
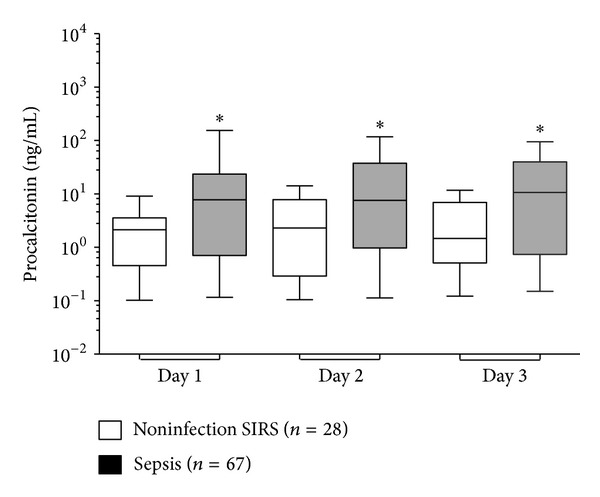
Daily concentration of PCT in sepsis and noninfectious SIRS. Boxes show the median and interquartile range, whiskers show 10–90th percentiles. *Mann-Whitney test between sepsis and noninfectious SIRS for procalcitonin, *P* = 0.02 (days 1 and 2) and *P* = 0.03 (day 3).

Ninety-five PCT determinations were made at baseline, 88 at 24 hours and 73 at 48 hours. PCT concentrations were consistently higher in sepsis compared to noninfectious SIRS throughout the 3-day period (Figure 1). Day 1-PCT concentration was associated with diagnosis of sepsis with an AUC of 0.65 (95% CI, 0.55 to 0.76, *P* = 0.02, *P* < 0.0001); however, it was not predictive of mortality with an AUC of 0.60 (95% CI, 0.46 to 0.74, *P* = 0.12).

#### 3.1.1. Predictive Performance of PCT in Subcohort of Patients with Severe Sepsis or Septic Shock

Sixty-seven patients had sepsis, of which all had at least one organ failure, and hence were described as having severe sepsis. Overall mortality was 40%, since 27 died in hospital. Day 1-PCT levels were much higher in the nonsurvivors than survivors, but it did not reach statistical significance (Table 2; *P* = 0.11). Both ΔPCT-24 and ΔPCT-48 were lower in nonsurvivors compared to survivors (*P* = 0.01 and 0.04, resp.).

**Figure 2:**
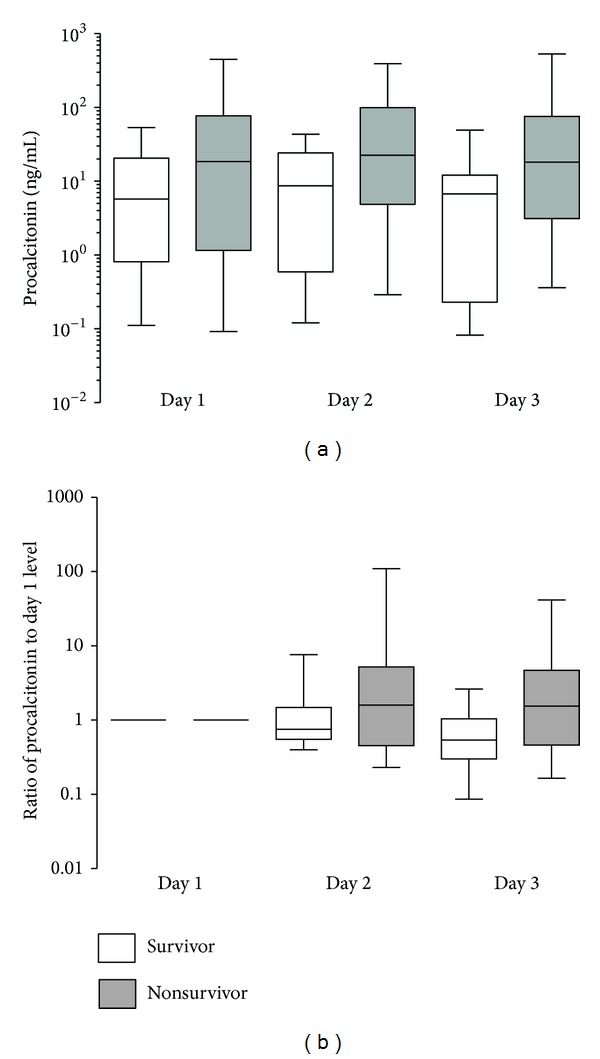
Temporal profiles of PCT in severe sepsis cohort between suvivors and nonsurvivors. (a) PCT concentration, Repeated Measures ANOVA (*P* = 0.012). (b) Ratio of PCT relative to Day 1.

The temporal profile of PCT between patients who survived and died is shown in Figure 2(a). The ratio of PCT on day 2 and day 3 relative to day 1 concentration is shown in Figure 2(b). PCT concentration showed a decay from baseline in the survivors, whereas it remained high in those who died.

#### 3.1.2. PCT Clearance

PCTc-48 was higher in patients who survived compared to those who died (Table 3; *P* = 0.02). PCTc-24 was also higher in survivors compared to nonsurvivors; however, it did not reach statistical significance (*P* = 0.10).

#### 3.1.3. PCT Clearance in Prediction of Survival

PCTc-48, but not PCTc-24, was associated with prediction of survival. The AUC for prediction of survival was 0.62 (0.46 to 0.78) for PCTc-24 and 0.69 (0.53 to 0.84) for PCTc-48. A cut-off point of 30% was used to stratify patients into those with PCTc-48 > 30% (Group 1) and those with PCTc-48 < 30% (Group 2). Hospital mortality was significantly lower in Group 1 compared with those in Group 2 (24.1% versus 52.6%, Chi-Square test, *P* = 0.02).

**Figure 3:**
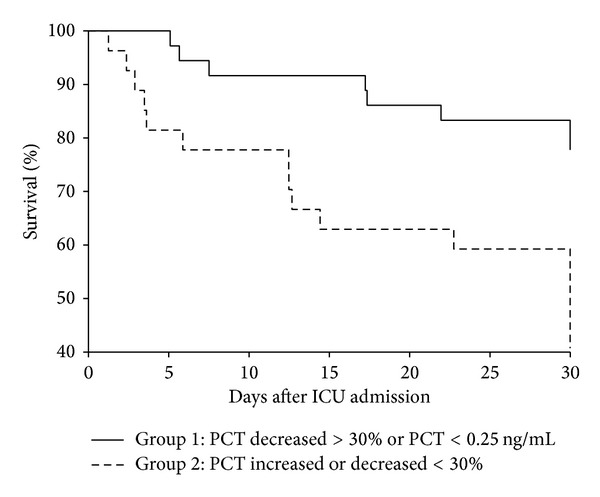
Kaplan Meier 30-day survival curve for groups 1 and 2. Log Mantel Cox Test (*P* = 0.002).

Survival analysis comparing Group 1 and Group 2 showed greater hospital mortality for Group 2 (log Mantel-Cox test, *P* = 0.002; Figure 3). After adjusting for severity of illness and organ failure (SAPS II and SOFA scores), Group 2 remained independently associated with mortality (HR 2.90 (95% CI 1.22 to 6.90)).

## 4. Discussion

In this prospective study of patients with SIRS, we demonstrated that serum PCT measured within 24 hours of ICU admission was associated with diagnosis of sepsis; however, it was not predictive of mortality. Our study showed that in a subcohort of patients with severe sepsis and septic shock, dynamic changes in PCT at 48 hours (PCT clearance) predicted survival. There was higher survival in septic patients with increased PCT clearance at 48 hours of more than 30% compared to those below 30%. The mean PCTc-24 was much higher in nonsurvivors but did not reach statistical significance.

In-hospital mortality rates for severe sepsis and septic shock are high, ranging between 30% and 50% [26, 27]. Malaysian Registry of Intensive Care 2012 reported sepsis as the first ranking diagnosis leading to ICU admission with 20.3% of ICU patients had severe sepsis during the first 24 hours. In their report, severe sepsis within 24 hours of admission carries in-hospital mortality of 43.1% and the observed mortality of all septic patients was 54.4% [28]. Therefore early risk stratification of septic patients that require intensification of therapy (e.g., early dialysis, modification of antimicrobial therapy, or the need for additional diagnosis measures) is required to prevent progression of organ failure and increased in mortality. The patients's clinical presentation may not always reflect the severity of sepsis syndrome.

We demonstrated that in patients with SIRS, PCT measured within 24 hours of ICU admission was associated with diagnosis of sepsis. This should prompt the clinician to search extensively for a source of sepsis and institute appropriate antibiotic treatment urgently. This is because time to antibiotic administration has been shown to be strongly associated with outcome [29]. Hence, early measurement of PCT concentration could guide clinicians in diagnosis of sepsis and early institution of a high quality and comprehensive therapy that may yield favourable outcome.

Once sepsis is diagnosed, prediction of survival is important for risk stratification of patients that may indicate the success or failure of the treatment. We evaluated the predictive performance of PCT in a subcohort of patients with severe sepsis or septic shock. Measurement of biomarker at a single time may be of limited value due to the great variability of biomarker secretion at different phases of critical illness and unknown time lapsed between the initiating insult and admission to the intensive care unit. Dynamic approach of assessing biomarkers captures the progression of the diseases and may be more helpful in managing critically ill patients. We showed that absolute level of early PCT measurement was not predictive of mortality; however, dynamic changes of PCT over 48 hours translated as PCT clearance were predictive. Therefore the prognostic significance of PCT may be apparent after determination of the progression of serial PCT concentrations relative to the baseline.

This supports the finding of other studies that demonstrated the prognostic utility of dynamic changes of PCT [18, 24, 30]. In a study of 37 septic shock patients with PCT concentration greater than 10 ng/mL, PCT decrease after 5 days to more than 25% of its baseline value is shown in all survivors and none in nonsurvivors [24]. In another larger study involving 180 septic patients, Day 3-PCT decrease of more than 30% from Day 2 (ΔPCT D2-D3) independently predicted survival, with an odds ratio of 2.94 (1.22 to 7.09) [30]. A recent retrospective study involving 156 sepsis patients in 2 ICU centres in the US showed that PCT changes within 72 critical care hours were independently associated with hospital mortality [31]. Additionally, we demonstrated the prognostic value of earlier PCT changes within the first 48 critical care hours in a more severe group of patients. The prospective nature of our study further adds to the strength of the finding.

The extent of changes in PCT may be influenced by its baseline concentration. Calculation of relative changes of PCT to the baseline concentration, or defined as PCT clearance, has been explored in a pilot study [18]. This can be used as a tool for monitoring the evolution of PCT levels during the course of severe sepsis. Ruiz-Rodriguez demonstrated that in 27 patients with septic shock, both PCT clearance at 24 and 48 hours were highly predictive of survival with AUC of 0.74 (95% CI, 0.54–0.950) and 0.86 (95% CI, 0.69–1.0), respectively. In another study involving 88 patients with septic shock, Suberviola et al. [19] showed that PCT clearance at 72 hours was higher in survivors compared to nonsurvivors and is predictive of survival with an AUC of 0.79. These 2 studies involved a high risk profile group of patients with septic shock. We demonstrated that in patients with severe sepsis and septic shock, PCT clearance at 48 hours was predictive of survival. In our study, only 43% of our patients had septic shock (57% severe sepsis) and hence represented a different severity from the other two studies.

The cut-off point of PCT clearance or its dynamic changes is of interest. This was recently investigated by Schuetz et al. [31] who showed that a cut-off point of 80% within 72 hours was associated with an abrupt increase in mortality (9.5% versus 47.8%) and had a high negative predictive value and sensitivity. In addition, Suberviola et al. [19] also showed an optimal cut-off point of 70% for PCTc-72 in predicting survival. In contrast, a lower cut-off point of 30% for PCT decrease within 24 hours, [30] and 48 hours [32] had been shown to be prognostic of survival. Since we assessed PCT dynamics at a shorter interval, that is, over 48 critical care hours, we chose 30% as our cut-off point. We showed that patients with 48-hour PCT clearance of more than 30% were 3 times more likely to survive compared to those with clearance of less than 30%. The prediction of hospital mortality for those with PCT increase or decrease <30% after 48 hours was independent of severity of illness as reflected by SOFA and SAPS II scores. From this, we suggest that PCT analysis should be measured on admission and repeated 48 hours later. PCT clearance of less than 30% indicates persistently elevated PCT levels and this could provide an early warning sign of patients who are at high risk of death and therefore should prompt the clinician to evaluate the “adequacy and appropriateness” [24] of therapy. Thus PCTc-48 could be used as risk stratification tool during reassessment of patients with severe sepsis and septic shock on day 3 of ICU stay. This prognostic tool may give some indication of possible modification of antimicrobial therapy, or the need for additional diagnosis measures, for example, a search for the focus of infection. Early identification of septic patients with a high-risk profile and provision of timely interventions could result in better outcomes. As such, there may be value in adding PCTc-48 analysis in patients with severe sepsis and septic shock, on top of the recommendations from guidelines such as the Surviving Sepsis Campaign.

*Study Limitations.* This study has several limitations. First, it was performed in a single centre and on small sample size. A multi-centre study involving larger patient population may confirm or refute the finding. Secondly, Day 1-PCT and PCTc-24 were much higher in the nonsurvivors compared to survivors; however, they were not statistically significant. This is because the study was not powered to detect the differences in Day 1-PCT or PCTc between survivors and nonsurvivors. Future larger study powered to look at this difference may refute or confirm the finding. Thirdly, the strict inclusion criteria of patients with severe sepsis in this study limit generalisability of the finding to other patient groups in the ICU. It would be interesting to look at the prognostic utility of PCT in other ICU patient groups as well. Fourthly, PCT was measured within 24 hours of starting antibiotic therapy and only daily afterwards. Blood sample taken within 2 to 4 hours after admission, and every 6 to 12 hours after admission up to 48 hours, would allow closer investigation of PCT profile and may provide an earlier point for optimal PCT clearance cut-off point. Fifthly, the study was underpowered to examine subgroup analyses and this may have affected the statistical results for such things as the PCTc-24. Finally, we did not collect daily SOFA score. This will enable comparison to PCT change, as change in SOFA has been shown to predict mortality risk in severe sepsis [33].

## 5. Conclusion

Early measurement of PCT is associated with diagnosis of sepsis in patients with SIRS. Dynamic changes of PCT reflected as PCT clearance after 48 hours from baseline are associated with prediction of survival in critically ill patients with severe sepsis. This could assist clinicians in risk stratification of critically ill patients with severe sepsis and septic shock. However, the small sample size and a single-centre study may limit the generalisability of the finding. This would benefit from replication in future multicentre study.

## Figures and Tables

**Table 1:** Baseline demographics, clinical characteristics, and outcomes between patients with SIRS and sepsis.

Variables	All patients (n = 95)	Noninfectious SIRS (n = 28)	Sepsis (n = 67)	*P*
Age (years)	44 ± 16	41 ± 17	45 ± 16	0.26
Gender (male)	66 (69.5)	19 (67.9)	47 (70.1)	0.83
Weight (kg)	67 ± 16	69 ± 22	66 ± 13	0.45
Height (cm)	159 ± 18	155 ± 31	161 ± 8	0.14
Baseline SAPS II Score	39 ± 16	33 ± 13	41 ± 16	0.02
Baseline SOFA Score	7.2 ± 4.3	5.2 ± 2.6	8.0 ± 4.7	0.003
Primary admission diagnoses				0.001
Cardiovascular	6 (6.3)	2 (7.1)	4 (6.0)	
Endocrine/metabolic	3 (28.6)	0 (0)	3 (4.5)	
Gastrointestinal/hepatobiliary/pancreas	7 (7.4)	1 (3.6)	6 (9.0)	
Infective	18 (18.9)	0 (0)	18 (26.9)	
Renal	4 (4.2)	2 (7.1)	2 (3.0)	
Neurological	12 (12.6)	3 (10.7)	9 (13.4)	
Respiratory	20 (21.1)	6 (21.4)	14 (20.9)	
Trauma	15 (15.8)	11 (39.3)	4 (6.0)	
Postoperative surgical	4 (4.2)	3 (10.7)	1 (1.5)	
Connective tissue/autoimmune	2 (2.1)	0 (0)	2 (3.0)	
Maternity/gynaecological	3 (3.2)	0 (0)	3 (4.5)	
Drug overdose/poisoning	1 (1.1)	0 (0)	1 (1.5)	
Renal replacement therapy (RRT)	28 (29.5)	4 (14.3)	24 (35.8)	0.04
Hospital mortality	30 (31.6)	3 (10.7)	27 (40.3)	0.008
Mechanical ventilation (MV)	87 (91.6)	25 (89.3)	62 (92.5)	0.60
Length of MV (days)	4.8 (1.8–9.9)	4.7 (1.0–9.5)	4.9 (2.1–13.0)	0.41
Length of ICU stay (days)	7.0 (2.9–14.6)	6.8 (2.7–10.3)	7.0 (3.2–15.8)	0.46
Length of hospital stay (days)	12.3 (6.6–18.2)	12.4 (6.4–17.4)	12.3 (6.6–19.2)	0.73

Data are expressed as mean ± SD, *n* (%), or median (lower quartile–upper quartile).

SAPS II Score: Simplified Acute Physiological II Score. SOFA Score: Sequential Organ Failure Assessment Score. SIRS: systemic inflammatory response syndrome.

**Table 2:** PCT as a function of survivors versus nonsurvivors in sepsis patients.

	Severe sepsis cohort (n = 67)	Survivors (n = 40)	Nonsurvivors (n = 27)	*P*
Day 1 PCT (ng/mL)	7.6 (1.0–37.7)	5.8 (0.8–20.6)	18.5 (1.1–77.2)	0.11
ΔPCT-24	0.03 (−6.2–6.7)	0.3 (−1.6–0.8)	−3.9 (−36–0.8)	0.01
ΔPCT-48	0.3 (−2.0–9.0)	0.8 (−0.01–12.4)	−1.4 (−17.2–2.3)	0.04

Data are presented as median (interquartile range). The minus sign indicates that the PCT level increased relative to the baseline PCT. Comparisons of the levels between survivors and nonsurvivors were analysed using Mann-Whitney test.

**Table 3:** PCT clearance in survivors and nonsurvivors.

	Sepsis cohort (*n* = 67)	Survivors (*n* = 40)	Nonsurvivors (*n* = 27)	*P*
		*n* = 39	*n* = 24	
PCTc-24	8.7 (−90.3–45.8)	24.7 (−48.2–45.0)	−60.3 (−423.2–54.6)	0.10

		*n* = 35	*n* = 20	
PCTc-48	31.6 (−67.1–68.7)	45.8 (−4.5–70.2)	−54.5 (−369.0–53.7)	0.02

Data are presented as median (interquartile range). The minus sign indicates that the PCT level increased relative to the baseline PCT. Comparisons of the levels between survivors and nonsurvivors were analysed with Mann-Whitney test.
